# Soluble programmed cell death-ligand 1 as a new potential biomarker associated with acute coronary syndrome

**DOI:** 10.3389/fcvm.2022.971414

**Published:** 2022-09-02

**Authors:** Shuping Li, Ling Yi, Xiqing Wei, Jinguo Zhang, Xiaojue Wang, Chang Jiang, Zhuohong Yan, Liwei Song, Bin Yang, Panjian Wei, Xiang Gao, Jinghui Wang, Hongtao Zhang, Jian Zhang

**Affiliations:** ^1^Beijing Tuberculosis and Thoracic Tumor Research Institute, Beijing Chest Hospital, Capital Medical University, Beijing, China; ^2^Department of Central Laboratory, Beijing Chest Hospital, Capital Medical University/Beijing Tuberculosis and Thoracic Tumor Research Institute, Beijing, China; ^3^Department of Cardiology, The Affiliated Hospital of Jining Medical University, Jining, China; ^4^Tumor Research Center, Beijing Chest Hospital, Capital Medical University/Beijing Tuberculosis and Thoracic Tumor Research Institute, Beijing, China; ^5^Department of Medical Oncology, Beijing Chest Hospital, Capital Medical University/Beijing Tuberculosis and Thoracic Tumor Research Institute, Beijing, China

**Keywords:** coronary artery disease, acute coronary syndrome, inflammatory biomarkers, high-sensitivity C-reactive protein, cytokines

## Abstract

**Background:**

Soluble programmed cell death-ligand 1 (sPD-L1) has been well documented to activate immunosuppression and is considered an essential predictor of negative clinical outcomes for several malignances and inflammatory conditions. However, the clinical significance of sPD-L1 in the peripheral blood of patients with coronary artery disease (CAD) remains unclear. The aim of this study was to assess the correlations of sPD-L1 with clinical features in CAD patients and evaluate the diagnostic value of this protein in CAD.

**Methods:**

A total of 111 CAD patients and 97 healthy volunteers who served as healthy controls (HCs) were consecutively enrolled. Plasma levels of sPD-L1 were measured with an amplified enzyme-linked immunosorbent assay (ELISA), and hs-CRP was measured with a C-reactive protein assay kit. The levels of other inflammatory cytokines were assessed in 88 CAD patients and 47 HCs by a multiparameter immunoluminescence flow cytometry detection technique. A logistic regression model was used to assess the independent association of sPD-L1 with acute coronary syndrome (ACS). The correlation between sPD-L1 and inflammatory cytokines in ACS was also assessed.

**Results:**

Plasma levels of sPD-L1 were significantly increased in CAD patients, especially those with ACS. Univariate logistic regression analysis revealed that sPD-L1 (OR: 3.382, 95% CI: 2.249–5.084, *p* < 0.001), BMI, hypertension, diabetes, dyslipidemia, previous MI, and the levels of HDL-C, LDL-C and hs-CRP were significantly associated with ACS. sPD-L1 (OR: 3.336, 95% CI: 1.084–6.167, *p* = 0.001) was found to be independently and significantly associated with ACS in the subsequent multivariable logistic regression analysis. Additionally, elevated plasma sPD-L1 levels were associated with increased interleukin-6 and interleukin-8 levels in ACS patients. Receiver operating characteristic (ROC) analysis showed that the AUC of sPD-L1 for diagnosing ACS was 0.778, with a sensitivity of 73.9% and a specificity of 73.4%, which was comparable with that of the inflammatory biomarker hs-CRP.

**Conclusion:**

The plasma sPD-L1 level reflects the severity of CAD, is associated with inflammatory responses and is a potential new biomarker for the diagnosis of ACS.

## Introduction

Coronary artery disease (CAD), a cardiovascular disease, is the leading cause of death in both developed and developing countries (1). CAD is an atherosclerotic disease (2) that manifests as chronic coronary syndrome (CCS), acute coronary syndrome (ACS) including acute myocardial infarction (AMI) and unstable angina (UA), or sudden cardiac death. We have gradually realized that inflammation may play a role in all stages of atherosclerosis (3). The results of recent clinical trials have firmly established that inflammation participates in the development of atherosclerotic events in humans (4). The recent Canakinumab Anti-inflammatory Thrombosis Outcomes Study (CANTOS) moved targeting inflammation in atherosclerosis from conjecture to clinical reality (5). This study targeted a specific proinflammatory cytokine, interleukin-1β (IL-1β), which has been implicated in atherogenesis over decades of experimental work. CANTOS showed that administration of an anti-IL-1β monoclonal antibody (mAb) to patients with stable cardiovascular disease following myocardial infarction who were treated according to current guidelines (including with statins) reduced the incidence of recurrent major adverse cardiovascular events. Subsequent clinical trials with colchicine, another anti-inflammatory agent, have likewise demonstrated a clinical benefit in patients with recent or temporally remote ACS (6, 7). Recently, in the RESCUE trial, the novel IL-6 ligand monoclonal antibody ziltivekimab was shown to be highly effective at reducing the levels of multiple inflammatory and thrombotic biomarkers relevant to atherosclerosis without the adverse effects associated with other agents in patients with chronic kidney disease whose high-sensitivity C-reactive protein (hs-CRP) level was at least 2 mg/L (8). These clinical trials have transformed the concept of inflammation in atherosclerosis from theory to practice. On the other hand, CRP and fibrinogen have been reported to be associated with an enhanced risk of stroke and coronary events in apparently healthy individuals (9, 10). Although inflammation has a central role in the pathogenesis of atherosclerosis, only a few related inflammation indicators can reflect the pathological process of atherosclerosis, especially in ACS.

Programmed death 1 (PD-1) is a key immunosuppressive molecule. PD-1 has two natural ligands, and its primary ligand, PD-L1 (B7-H1, CD274), is expressed on the surface of activated T cells and macrophage-lineage cells, as well as some non-hematologic cells, such as those in the placenta and heart (11, 12). Tumor cells can also express PD-L1 through oncogene activation as a tumor immune escape mechanism. Thus far, PD-L1 has been proven to play a major role as a negative regulator of antitumor immunity, and blockade of the PD-1/PD-L1 interaction permits restoration and enhancement of the function of T cells. Targeting the PD-1/PD-L1 pathway in diverse tumors has achieved great clinical success (13, 14).

As a transmembrane protein, PD-L1 is mainly expressed on the cell surface. In addition, several soluble forms of PD-L1 (sPD-L1) have been described, including cleaved, alternatively spliced and exosomal sPD-L1, which are released from cells into body fluids. Similar to membrane-bound PD-L1, sPD-L1 is currently thought to contribute to systemic immunosuppression (15–17). Several published reports have shown that sPD-L1 is an indispensable predictive factor in various types of cancer, especially for checkpoint blockade treatments (17–23). Moreover, sPD-L1 production has been reported to be increased in many other diseases, including chronic inflammation and viral diseases, and under non-pathological conditions, such as pregnancy (15, 24). Recently, it was reported that PD-1/PD-L1 immunotherapy increased the risk of atherosclerosis-related cardiovascular disease among patients with cancer and that the immune response of the PD-1/PD-L1 pathway in cardiovascular disease is related to the severity of atherosclerosis (25). These clinical presentations imply that the PD-1/PD-L1 pathway may be related to the inflammatory pathology of atherosclerosis. Therefore, we speculated that sPD-L1 is a potentially relevant biomarker in the blood of CAD patients. In this study, we aimed to elucidate these issues via the following clinical evaluations: (a) determining the correlations of plasma levels of sPD-L1 with the severity of CAD, (b) exploring the association of plasma sPD-L1 with blood-based inflammation markers such as hs-CRP and a panel of other cytokines, and (c) evaluating the predictive value of sPD-L1 for ACS.

We found that plasma levels of sPDL-1 were definitely increased in CAD, particularly in ACS patients. The production of sPD-L1 tends to reflect the severity of CAD disease and is associated with inflammatory responses in atherosclerosis. These preliminary results may provide clues for developing new biomarkers for CAD patients in clinical practice.

## Materials and methods

### Study subjects

One hundred thirty-three CAD patients who were screened for coronary disease by coronary angiography were consecutively admitted to the Beijing Chest Hospital Heart Center and Emergency Department at the Affiliated Hospital of Jining Medical University between February 2021 and November 2021. The exclusion criteria included malignant diseases, current hemodialysis, active systemic inflammatory diseases (autoimmune diseases, rheumatoid diseases requiring immunosuppressive therapies, and infectious diseases), and peripheral arterial occlusive diseases causing pain at rest during the same study period. In addition, 97 of 127 healthy volunteers were included as the control (HC) group. Ultimately, 111 patients and 97 HCs were included in this study. The studies involving human participants were reviewed and approved by the Ethics Committee of Beijing Chest Hospital, Capital Medical University (IRB number: 2021-KY-35) and the Affiliated Hospital of Jining Medical University (2022C047) and were performed according to the Declaration of Helsinki Principles.

### Definition of acute myocardial infarction, unstable angina, acute coronary syndrome, chronic coronary syndrome, and coronary artery disease

Acute myocardial infarction (AMI) was defined as an increase in plasma levels of cardiac troponin above the 99th percentile of the upper limit of the normal range together with evidence of myocardial ischemia and at least one of the following findings: electrocardiographic changes (new ST-T changes, left bundle branch block, or pathologic Q wave), imaging evidence of new viable myocardial loss, or a new abnormality in regional wall motion (26). Unstable angina (UA) was defined as new or accelerated symptoms of myocardial ischemia accompanied by new ischemic ST-T-wave changes. ACS included AMI and UA. Chronic coronary syndrome (CCS) was defined as a history of angina pectoris or myocardial ischemia by stress tests coupled with coronary stenosis of >50% of the vessel diameter detected by coronary angiography and/or a history of myocardial infarction, percutaneous coronary intervention (PCI), or coronary artery bypass grafting (CABG). Therefore, all of the conditions described above were defined as CAD. This study also excluded patients with CAD caused by coronary vasospasm. All patients participating in the study met the diagnostic criteria for CAD set by the American College of Cardiology Foundation/American Heart Association (ACC/AHA).

### Blood sampling

Peripheral blood samples of patients were obtained in the catheter-laboratory room soon after sheath insertion and before the administration of heparin. Clinical parameters (hemoglobin, HbA1c, triglycerides, HDL-C, LDL-C, ejection fraction, creatinine and BNP) were part of the standard workup at the Laboratory Diagnosis Center. In addition, to measure the levels of sPD-L1, high-sensitivity troponin I (hs-TnI), hs-CRP and inflammatory cytokines under the same conditions, all blood samples, including those from the 68 AMI, 24 UA, and 19 CCS patients and the 97 HCs, were centrifuged at 2500 rpm for 10 min at room temperature, and the plasma was collected and kept frozen at −80°C until measurement. Repeated cycles of freezing and thawing of the samples were avoided.

### Generation of programmed cell death ligand-1 monoclonal antibodies and establishment of sandwich enzyme-linked immunosorbent assay

Monoclonal anti-human PD-L1 mAbs were generated by immunizing BALB/c mice with PD-L1-Fc fusion proteins containing the extracellular domain (Met 1-Thr 239) of human PD-L1 (B7-H1, NP_054862.1) with the C-terminal fused Fc region of human IgG1 that was expressed in HEK293 Cells. Using immune spleen cells and the mouse myeloma SP2/0 cell line as a fusion partner, mouse hybridomas were generated and selected by the same PD-L1-Fc fusion protein and PD-L1-His (Met 1-Thr 239) fusion protein. More than 40 positive wells were screened, and two mAbs, 11E3 and 2F1, were finally identified. The two antibodies were effectively used in ELISA, flow cytometry and western blot, and 11E3 can also be used in immunohistochemical detection. In addition, 11E3 is the blocking antibody of PD-1/PD-L1 binding, while 2F1 is the non-blocking antibody. Therefore, the two antibodies were successfully paired for sandwich ELISA. 11E3 was used as the capture antibody, and biotin-labeled 2F1 was used as the detection antibody, so an amplification test with an expected sensitivity of 5 pg/ml (*R*^2^ = 0.9998) was established.

### Plasma soluble programmed cell death-ligand 1 detection

We measured plasma levels of sPD-L1 in all 111 CAD patients and 97 healthy controls by sandwich ELISA. A standard curve was generated for each assay using recombinant human PD-L1-His with a purity >98% (Sino Biological Inc., Beijing, China). Plasma levels of sPD-L1 were measured using ELISA Costar ELISA plates coated with anti-human PD-L1 mAb (2 μg/ml, 11E3) overnight at 4°C. The plates were then blocked with 5% skim milk (BD Difco, Sparks, MD, USA) for 2 h 37°C. Plasma samples that were obtained from patients and healthy controls and diluted 25-fold were added to each well of the antibody-coated plates and incubated overnight at 4°C. The captured sPD-L1 was detected using 2 μg/ml biotinylated anti-PD-L1 mAb (Bio2F1) followed by incubation with streptavidin-HRP (1:2000, Cell Signaling Technology, Danvers, MA, USA) for 0.5 h at room temperature (RT). The samples were developed using the TMB Substrate Reagent Set (BD OptEIA, San Diego, CA, USA), and then Stop Solution (BD OptEIA, San Diego, CA, USA) was added. The optical density (OD) was evaluated at 450 nm with a MULTISKAN GO instrument (Thermo Scientific, Waltham, MA, USA). The plates were washed three times with 1 × PBS containing 0.5‰ Tween-20 following every step.

### Measurement of plasma levels of high-sensitivity C-reactive protein, high-sensitivity troponin I, and cytokines

According to the Centers for Disease Control and Prevention and the American Heart Association, it is recommended that hs-CRP be monitored to assess cardiovascular risk and tested as a routine laboratory parameter (27). Hs-CRP was adopted as the assessment index for disease association in this study. It has been reported that the new full-range Olympus assay can be effectively used to measure hs-CRP as a marker of inflammation and cardiovascular risk (28). Therefore, we measured the levels of hs-CRP from the same plasma samples used to quantify the levels of sPD-L1 by using CRP Latex (Beckman Coulter, Inc., Brea, CA, USA) according to the manufacturer’s instructions. This assay uses an immunoturbidimetric test for quantification of hs-CRP. The test is linear on the AU640 system within the concentration ranges from 0.2 to 480 mg/L for normal application and 0.08–80 mg/L for highly sensitive application. In addition, the plasma levels of hs-TnI were measured in 111 CAD patients and 10 of 97 HCs (randomly selected) by an AQT90 FLEX TnI Test Kit (Radiometer Medical ApS, Turku, Finland).

To further evaluate the systemic inflammatory response in patients with ACS, we randomly selected 88 of the 111 CAD patients and 47 of the 97 HCs for the measurement of 14 inflammatory cytokines. These selected plasma cytokines were measured by an immunofluorescence assay that can recognize 14 cytokines (IFN-γ, IL-1β, IL-2, IL-4, IL-5, IL-6, IL-8, IL-10, IL-12p70, IL-17, and TNF-α) (Quantobio, Beijing, China) according to the manufacturer’s instructions. The assay is based on the principle of combining a liquid-phase chip and double antibody sandwich and consists of 14 kinds of microspheres with different fluorescence intensities coated with specific antibodies for each cytokine (capture microspheres), which can capture the corresponding cytokines in plasma samples. When the corresponding cytokine is specifically bound, a biotin-labeled detection antibody is added to form an immune complex of capture microspheres and cytokine-detection antibody; then, PE-labeled streptavidin (SA-PE) is added, and the corresponding fluorescence on the microspheres is detected using multiparameter immunoluminescence flow cytometry (BD LSRFortessa, San Jose, CA, USA). Thus, the concentration of the individual cytokines in the sample to be measured is obtained from the fluorescence intensity of the immune complex microspheres. The relative deviation of the detection results is within ±15%, and the lower limit of detection (LOD) of this kit is 2.3 pg/mL.

### Statistical analysis

Continuous variables were expressed as the mean ± standard deviation (SD) for normally distributed variables according to the Shapiro-Wilk test, and the median value with interquartile range (IQR) was used for a non-normal distribution. Categorical variables were presented as frequencies and percentages. The χ^2^ or Fisher’s exact test was used to compare differences between categorical variables. The Bonferroni method was used to adjust for comparisons among three or more groups. The Mann–Whitney test was used for two-group comparisons. The Kruskal–Wallis test was used for non-normal distributions to compare three or more groups. Clinical parameters that were significantly associated with ACS in univariate logistic regression analysis were evaluated using multivariable logistic regression analysis. The odds ratios (ORs) and 95% confidence intervals (CIs) were presented. *Z* scores were also used to make ORs comparable (per standard deviation) among the biomarker covariates in logistic regression analyses. To assess model calibration, the Hosmer–Lemeshow statistic for multivariable logistic regression was applied. To compare the association, Spearman’s rank correlation coefficient was calculated between each data set. Receiver operating characteristic (ROC) analysis was applied to evaluate the accuracy and diagnostic ability of sPD-L1. The area under the curve (AUC) of the ROC curve was calculated, along with sensitivity and specificity values. A *p*-value < 0.05 was considered indicative of statistical significance. Statistical analyses were performed using SPSS software v.20.0 (IBM Corp., Armonk, NY, USA). The statistical software GraphPad Prism 8.0 (Graph-Pad Software, La Jolla, CA, USA) was used for all statistical tests and graph creation.

## Results

### Study subjects and baseline clinical characteristics

**FIGURE 1:**
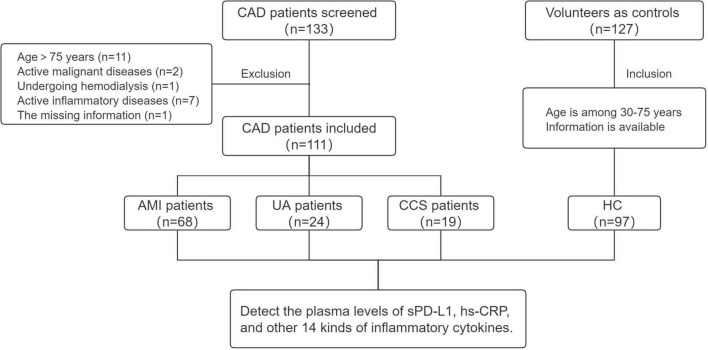
Study flow chart. CAD, coronary artery disease; AMI, acute myocardial infarction; UA, unstable angina; CCS, chronic coronary syndrome; HC, healthy control.

A total of 133 patients with CAD who were screened for coronary disease by coronary angiography (coronary stenosis of >50% of the vessel diameter detected) and/or a history of MI, PCI, or CABG were consecutively admitted to the Heart Center of Beijing Chest Hospital, Capital Medical University and the Affiliated Hospital of Jining Medical University between February 2021 and November 2021. Among them, 22 patients met the following exclusion criteria: active malignant diseases (*n* = 2), current hemodialysis (*n* = 1), active systemic inflammatory diseases (autoimmune diseases and rheumatoid diseases requiring immunosuppressive therapies) (*n* = 7), age greater than 75 years (*n* = 11), and missing information (*n* = 1). One hundred twenty-seven healthy volunteers were included as the healthy control (HC) group. Finally, 111 patients with CAD (AMI, *n* = 68; UA, *n* = 24; CCS, *n* = 19) and 97 HCs were enrolled in this study. The study flow chart is presented in Figure 1.

The baseline characteristics of all the enrolled study subjects are summarized in Table 1. The clinical parameters age, sex, BMI, previous MI, family history, hypertension, diabetes, dyslipidemia, current smoking, HbA1c, BNP, creatinine, triglycerides, HDL-C, aspirin, stains, and CCBs were similar among the ACS and CCS groups. Compared with CCS patients, ACS patients showed significantly higher levels of LDL-C [(2.66 (2.16–3.48) vs. 2.01 (1.62–2.84) mg/dL, *p* = 0.017], hemoglobin [(138.42 ± 15.39) vs. (129.11 ± 14.26) g/dL, *p* < 0.001], ejection fraction [0.53 (0.46–0.58) vs. 0.62 (0.59–0.63), *p* < 0.001], hs-TnI [0.62 (0.328–5.308) vs. 0 (0–0.03) ng/ml, *p* < 0.001], nitrate use [54 (58.7) vs. 5 (26.3), *p* = 0.01], beta blocker use [74 (80.4) vs. 10 (52.6), *p* = 0.024], ACEI or ARB use [57 (62) vs. 4 (21.1), *p* < 0.001], and hs-CRP [1.71 (0.96–4.25) vs. 0.15 (0.14–0.23), *p* < 0.001] (Table 1). Compared with the HCs, CAD patients and had higher BMI, triglyceride levels, hs-CRP levels and rates of previous MI, hypertension, diabetes, and dyslipidemia; moreover, they had lower levels of HDL-C, hemoglobin (Supplementary Table S1).

**TABLE 1:** Baseline characteristics of 111 enrolled patients.

Variable	ACS	CCS	HC	*P*-value
			
	*N* = 92	*N* = 19	*N* = 97	
Age, years	58.85 ± 11.42*	59.11 ± 12.76*	55.21 ± 13.8	0.206
Male, *n* (%)	63 (63.8)*	11 (57.9)*	69 (71.1)	0.522
BMI (kg/m^2^)	26.05 ± 3.99*	24.96 ± 5.97	24.54 ± 3.04	0.01
Current smoking, *n* (%)	45 (48.9)	12 (63.2)	—	0.258
Family history, *n* (%)	10 (10.9)	2 (10.5)	—	1
Previous MI, *n* (%)	8 (8.7)*	1 (5.3)*	0 (0)	0.015
Hypertension, *n* (%)	68 (73.9)*	18 (94.7)*	0 (0)	<0.001
Diabetes, *n* (%)	32 (34.8)*	6 (31.6)*	0 (0)	<0.001
Dyslipidemia, *n* (%)	55 (59.8)*	11 (57.9)*	33 (34)	0.001
**Medication**				
Aspirins, *n* (%)	92 (100)	18 (94.7)	—	0.171
Statins, *n* (%)	88 (95.7)	17 (89.5)	—	0.202
Nitrates, *n* (%)	54 (58.7)^#^	5 (26.3)	—	0.01
Beta blockers, *n* (%)	74 (80.4)^#^	10 (52.6)	—	0.024
CCBs, *n* (%)	13 (14.1)	6 (31.6)	—	0.133
ACEI/ARBs, *n* (%)	57 (62)^#^	4 (21.1)	—	<0.001
Creatinine, μmol/L	65.7 (57.53–76.78)	69.2 (57.8–79.9)	67 (58.6–75)	0.646
Triglycerides, mg/dL	1.53 (1.06–2.19)	1.57 (1.09–2.11)	1.29 (0.94–1.78)	0.078
HDL-C, mg/dL	1.13 (0.98–1.28)*	1.05 (0.95–1.13)*	1.49 (1.16–1.71)	<0.001
LDL-C, mg/dL	2.66 (2.16–3.48)^#^	2.01* (1.62–2.84)*	2.67 (2.25–2.98)	0.017
Hemoglobin, g/dL	138.42 ± 15.39^#^	129.11 ± 14.26*	143.4 ± 13.76	0.002
HbA1c, %	6.15 (5.70–7.15)	6.2 (5.4–9.2)	—	0.811
BNP, pg/mL	145 (75–449.94)	109 (70–261.5)	—	0.308
Ejection fraction, %	0.53 (0.46–0.58)^#^	0.62 (0.59–0.63)	—	<0.001
hs-TnI, ng/mL	0.62 (0.328–5.308)^#^	0 (0–0.03)	—	<0.001
hs-CRP, mg/L	1.71 (0.96–4.25)*	0.15 (0.14–0.23)*	0.68 (0.42–1.57)	<0.001
sPD-L1, pg/mL	256.72 (204.57–301.01)^#^*	188.32 (160.67–216.82)	173.06 (144.81–231.50)	<0.001

Data are presented as the n (%), mean ± SD, or median (IQR). ACS, acute coronary syndrome; CCS, chronic coronary syndrome; HC, healthy control; CCB, calcium channel blockers; HDL-C, high-density lipoprotein-cholesterol; LDL-C, low-density lipoprotein-cholesterol; HbA1c, hemoglobin A1c, BNP, B-type natriuretic peptide; hs-CRP, high-sensitivity C-reactive protein; hs-TnI, high-sensitivity troponin I; sPD-L1, soluble programmed cell death ligand-1.

^#^p < 0.05 compared with CCS and *p < 0.05 compared with HC.

### Associations between routine clinical factors, biomarkers and soluble programmed cell death-ligand 1 with acute coronary syndrome

The results of univariate and multivariate logistic regression analyses by absolute concentration values and by transformation of biochemical covariates to *Z* scores are summarized in Table 2. Univariate logistic regression analysis identified that routine clinical factors, including BMI, hypertension, diabetes, dyslipidemia, previous MI and the levels of HDL-C, LDL-C and hs-CRP, were significantly associated with ACS. These significant parameters, identified from the univariate analysis, were further included in the multivariate logistic regression model. As indicated in Table 2, there was only one routine clinical factor, hypertension (OR: 19.19, 95% CI: 6.613–59.758, *p* < 0.001), that was significantly associated with ACS. In addition, one laboratory index, hs-CRP (OR: 10.94, 95% CI: 2.97–40.313, *p* < 0.001), was significantly and independently associated with ACS. The Hosmer–Lemeshow goodness of fit chi-square result was 3.086 with a *p* value of 0.929. Relying on the same analysis method, this study found that sPD-L1 was also significantly associated with ACS by univariate logistic regression analyses (OR: 3.382, 95% CI: 2.249–5.084, *p* < 0.001). Moreover, the multivariate logistic regression analyses that included nine significant factors identified in the univariate analysis demonstrated that sPD-L1 was significantly and independently associated with ACS (OR: 3.336, 95% CI: 1.804–6.167, *p* < 0.001), which indicated that sPD-L1 could be a new laboratory biomarker that is independently associated with ACS (Table 2).

**TABLE 2:** Logistic regression analyses using *Z* score for acute coronary syndrome.

Variable	Univariate			Multivariable		
		
	OR	95% CI	*P*-value	OR	95% CI	*P*-value
Z score_Age, per 1	1.271	0.961–1.682	0.093	—	—	—
Male, yes	0.978	0.542–1.764	0.94	—	—	—
Z score_BMI, per 1	1.474	1.102–1.972	0.009	1.132	0.697–1.838	0.615
Hypertension, yes	24.556	11.514–52.369	<0.001	19.19	6.163–59.758	<0.001
Dyslipidemia, yes	2.5	1.418–4.409	0.002	1.413	0.539–3.705	0.482
Diabetes, yes	9.778	3.87–24.706	<0.001	1.336	0.320–5.577	0.691
Previous MI, yes	10.952	1.344–89.243	0.025	3.184	0.295–34.32	0.34
Current smoking, yes	0.559	0.202–1.546	0.262	—	—	—
Z score_BNP, per 1	1.837	0.624–5.409	0.270	—	—	—
Z score_Creatinine, per 1	1	0.76–1.317	0.998	—	—	—
Z score_Hemoglobin, per 1	0.84	0.636–1.11	0.205	—	—	—
Z score_HDL-C, per 1	0.452	0.321–0.635	<0.001	0.669	0.377–1.185	0.168
Z score_LDL-C, per 1	1.365	1.019–1.827	0.037	1.627	0.96–2.759	0.071
Z score_Triglycerides, per 1	0.143	0.93–1.649	0.718	—	—	—
Z score_HbA1c, per 1	0.256	0.406–1.271	0.269	—	—	—
Z score_hs-TnT, per 1	4.83	0.521–44.783	0.087	—	—	—
Statins, yes	1.222	0.129–11.587	0.861	—	—	—
Z score_hs-CRP, per 1	7.988	3.176–20.088	<0.001	10.94	2.97–40.313	<0.001
Z score_sPD-L1, per 1	3.382	2.249–5.084	<0.001	3.336	1.804–6.167	<0.001

OR, odds ratio; CI, confidence interval. See Table 1 for other abbreviations.

The Hosmer–Lemeshow goodness of fit chi-square result was 3.086 with a p-value of 0.929.

### Plasma levels of soluble programmed cell death-ligand 1 are upregulated during coronary artery disease and tend to reflect the severity of the disease

**FIGURE 2:**
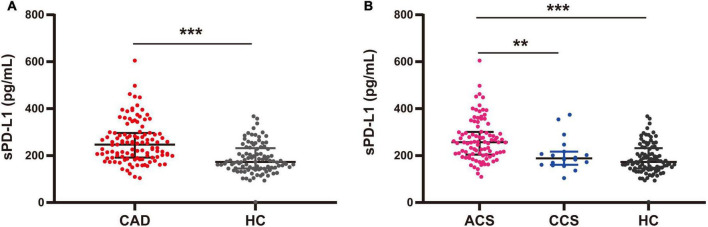
Plasma levels of sPD-L1 among CAD, ACS, and CCS patients and HCs. **(A)** Comparison of sPD-L1 levels between CAD patients (*n* = 111) and HCs (*n* = 97). **(B)** Comparison of sPD-L1 levels among ACS patients (*n* = 92), CCS patients (*n* = 19), and HCs (*n* = 97). There was no age difference between the groups. **p < 0.01; ***p < 0.001. sPD-L1, soluble form of programmed cell death ligand-1; CAD, coronary artery disease; ACS, acute coronary syndrome; CCS, chronic coronary syndrome; HCs, healthy controls.

We then assessed the differences in sPD-L1 levels in peripheral blood among the various study cohorts. Initially, we measured the plasma sPD-L1 levels in 97 HCs at a median level of 173.06 (144.81–231.50) pg/ml, and in 111 CAD patients at the median level of 247.03 (191.86–296.34) pg/ml. In addition, we analyzed the ACS and CCS subgroups from the overall group of 111 CAD patients. The median levels of sPD-L1 were 256.72 (204.57–301.01) and 188.32 (160.67–216.82) pg/ml, respectively. Statistically, patients with CAD showed significantly higher levels of sPD-L1 than HCs [CAD, 247.03 (191.86–296.34) vs. HC, 173.06 (144.81–231.50) pg/ml, *p* < 0.001] (Figure 2A and Supplementary Table S1). Furthermore, the levels of sPD-L1 were significantly higher in patients with ACS than in those with CCS [ACS, 256.72 (204.57–301.01) vs. CCS, 188.32 (160.67–216.82) pg/ml, *p* = 0.006] and HCs [173.06 (144.81–231.50) pg/ml, *p* < 0.001] (Table 1 and Figure 2B). The sPD-L1 level was age dependent, but the level of sPD-L1 between the CAD and age-matched HC groups was still significantly different (*p* < 0.001) (Supplementary Figure S1).

### Analysis of the association between plasma soluble programmed cell death-ligand 1 and myocardial injury in patients with acute coronary syndrome

**FIGURE 3:**
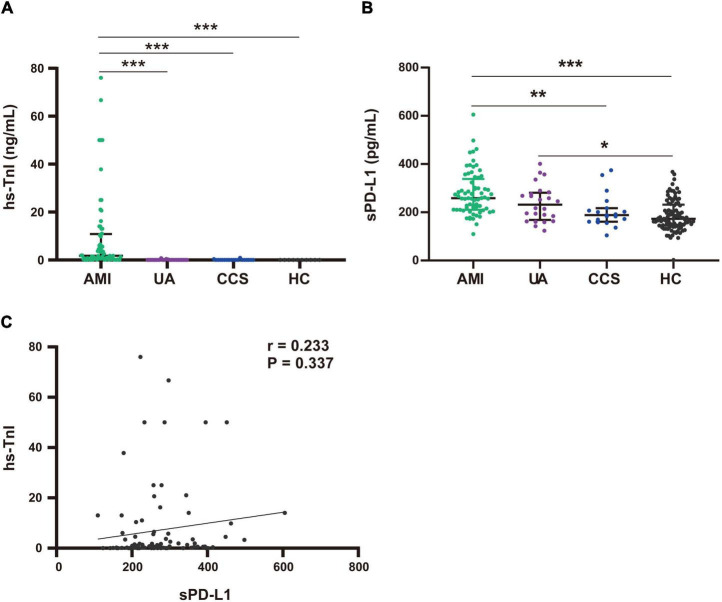
Comparison of plasma hs-TnI and sPD-L1 levels among the AMI, UA, CCS and HC groups. **(A)** Comparison of hs-TnI levels among the AMI (*n* = 68), UA (*n* = 24), CCS (*n* = 19), and HC (*n* = 10) groups. **(B)** Comparison of sPD-L1 levels among the AMI (*n* = 68), UA (*n* = 24), CCS (*n* = 19), and HC (*n* = 97) groups. **(C)** Association analysis of sPD-L1 with hs-TnI in the ACS group (*n* = 92). *p < 0.01, **p < 0.01, ***p < 0.001. hs-TnI, high-sensitivity troponin I; sPD-L1, soluble programmed cell death ligand-1.

To explore whether the source of plasma sPD-L1 may be related to cardiomyocyte injury, we first assessed the differences in hs-TnI levels among patients in the AMI, UA, CCS and HC groups. The levels of hs-TnI were significantly higher in patients with AMI than in those with UA [AMI, 1.63 (0.32–10.84) ng/ml vs. UA, 0.005 (0.001–0.275) ng/ml, *p* < 0.001], those with CCS [0.001 (0.001–0.03) ng/ml, *p* < 0.001], and the HCs [0 (0–0) ng/ml, *p* < 0.001) (Figure 3A). There was no significant difference in hs-TnI levels between the UA and HC groups [UA, 0.0 (0–0.275) vs. HC, 0 (0–0) ng/ml, *p* = 1] (Figure 3A). In contrast, in this study, the plasma levels of sPD-L1 were higher in UA patients than in HCs [UA, 231.3 (168.41–282.15) vs. HC, 173.06 (144.81–231.50) pg/ml, *p* = 0.021] (Figure 3B). These results indicated that the role of sPD-L1 in the development of CAD seems to be different from that of hs-TnI, which has now become widely used as the gold standard for identifying patients with AMI (29). Additionally, further analysis failed to yield any significant association of plasma sPD-L1 with plasma hs-TnI in patients with ACS (*r* = 0.233, *p* = 0.337) (Figure 3C). These results did not indicate an internal relationship between plasma sPD-L1 and myocardial injury.

### Elevated plasma soluble programmed cell death-ligand 1 levels are associated with increased systemic inflammatory cytokine interleukin-6 and interleukin-8 levels in patients with acute coronary syndrome

**FIGURE 4:**
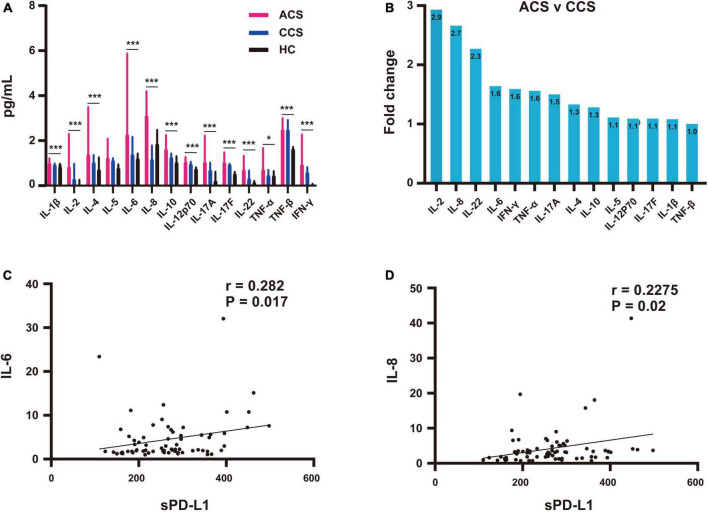
Association of plasma sPD-L1 with elevated IL-6 and IL-8 levels in ACS patients. **(A)** Comparison of elevated fold changes in inflammatory cytokine levels among the ACS (*n* = 71), CCS (*n* = 17) and HC (*n* = 47) groups. **(B)** Increased inflammatory cytokine concentrations in the ACS group compared with the CCS group. **(C)** Association of sPD-L1 with IL-6 in the ACS (*n* = 71) group. **(D)** Association of sPD-L1 with IL-8 in the ACS (*n* = 71) group. *p < 0.05; ***p < 0.001.

We also analyzed the association between plasma inflammatory cytokines and sPD-L1 levels. We used the multiparameter immunoluminescence flow cytometry detection technique and double antibody sandwich assay to measure the concentrations of 14 plasma cytokines, including IFN-γ, IL-1β, IL-2, IL-4, IL-5, IL-6, IL-8, IL-10, IL-12p70, IL-17A, IL-17F, IL-22, TNF-α and TNF-β, in 88 of the 111 patient samples and 47 of the 97 HC samples. All the cytokines were higher in the CAD patients than in the HCs (Supplementary Table S2). We then compared the ACS group with the CCS and HC groups, and ACS patients showed significantly higher levels of the 14 inflammatory cytokines (Table 3 and Figure 4A). Among these cytokines with increased concentrations, the leading cytokines with a fold change (FC) increase ≥ 1.5 times in plasma were IL-2, IL-8, IL-22, IL-6, IFN-γ, TNF-α and IL-17A, which showed significantly higher levels in ACS patients than in CCS patients (Figure 4B). However, by further analyzing the associations between all the inflammatory cytokines above with increased levels and sPD-L1 in ACS patients, we found that only the plasma levels of IL-6 and IL-8 were significantly associated with the sPD-L1 level, even though the correlations were relatively weak [IL-6, (*r* = 0.282, *p* = 0.017); IL-8, (*r* = 0.2275, *p* = 0.02)] (Figures 4C,D). In addition, an increased IFN-γ level was associated with sPD-L1 levels in UA patients (*r* = 0.55, *p* = 0.008) (Supplementary Figure S2). These results support that sPD-L1 is likely to be a biomarker related to the inflammatory response in patients with ACS.

**TABLE 3:** The plasma levels of inflammatory cytokines in 88 patients.

Cytokines	ACS	CCS	HC	*P*-value
			
(pg/mL)	*N* = 71	*N* = 17	*N* = 47	
IL-1β	0.98 (0.86–1.21)^#^*	0.91 (0.86–0.99)	0.82 (0.61–0.95)	<0.001
IL-2	0.82 (0–2.29)*	0.28 (0–0.97)*	0 (0–0.25)	<0.001
IL-4	1.36 (0.85–3.49)*	1.02 (0.89–1.36)	0.70 (0.31–1.26)	<0.001
IL-5	1.22 (0.96–2.09)*	1.10 (0.82–1.22)	0.77 (0.59–0.95)	<0.001
IL-6	2.25 (1.74–5.87)*	1.37 (1.2–2.16)*	1.19 (0.91–1.42)	<0.001
IL-8	3.09 (1.59–4.19)^#^*	1.16 (1.05–1.78)	1.85 (1.13–2.48)	<0.001
IL-10	1.60 (1.30–2.24) ^#^*	1.25 (1.08–1.43)*	1.02 (0.82–1.31)	<0.001
IL-12p70	1.04 (0.91–1.27)^#^*	0.95 (0.8–1.06)*	0.74 (0.63–0.82)	<0.001
IL-17A	1.02 (0.53–2.22)*	0.68 (0.4–1.02)*	0.2 (0–0.62)	<0.001
IL-17F	1.01 (0.73–1.48)*	0.93 (0.81–0.97)*	0.50 (0.43–0.61)	<0.001
IL-22	0.68 (0.35–1.32)*	0.3 (0.26–0.67)*	0.13 (0.06–0.21)	<0.001
TNF-α	0.70 (0.28–1.67)*	0.45 (0.27–0.7)*	0.43 (0.3–0.64)	0.014
TNF-β	2.47 (2.11–3.0)*	2.47 (2.05–2.92)*	1.62 (1.52–1.72)	<0.001
IFN-γ	0.92 (0.28–2.27)*	0.58 (0.22–0.82)*	0 (0–0.08)	<0.001

Data are presented as the median (IQR). ACS, acute coronary syndrome; CCS, chronic coronary syndrome; HC, healthy control; IL, interleukin; TNF-α, tumor necrosis factor alpha; TNF-β, tumor necrosis factor beta; IFN-γ, interferon gamma.

^#^p < 0.05 compared with CCS and *p < 0.05 compared with HC.

### Receiver operating characteristic analysis to evaluate the diagnostic accuracy of soluble programmed cell death-ligand 1 for acute coronary syndrome

**FIGURE 5:**
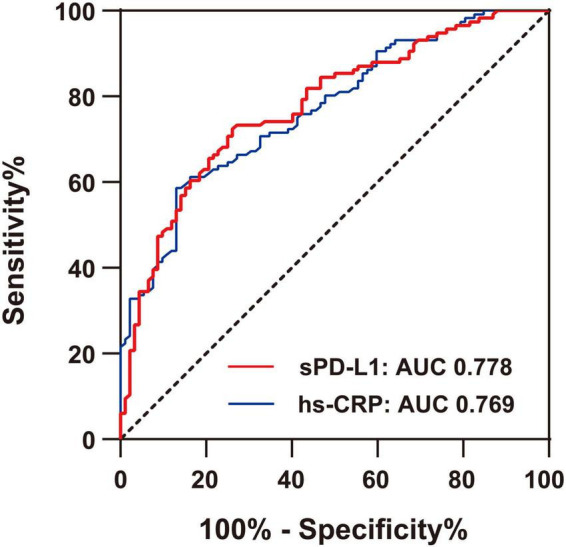
Comparison of ROC curves for the diagnostic values of hs-CRP and sPD-L1 in ACS. The area under the curve (AUC) of sPD-L1 and hs-CRP in ACS (*n* = 92) was indicated, with a sensitivity of 73.9% and a specificity of 72.4% for sPD-L1 and a sensitivity of 79.3% and a specificity of 72.1% for hs-CRP.

In this study, sPD-L1 was shown to be an independent predictive marker associated with ACS. Finally, we evaluated its diagnostic accuracy for ACS patients. As indicated in Figure 5, the AUCs generated by ROC analysis showed the diagnostic efficacies of sPD-L1 and hs-CRP in the same population of ACS patients. The optimum sPD-L1 cutoff for the diagnosis of ACS was 208.21 pg/ml, with a sensitivity of 73.9% and a specificity of 72.4%. The area under the curve (AUC) was 0.778 (95% CI: 0.715–0.841, *p* < 0.001), which showed a favorable diagnostic efficacy for ACS. In addition, hs-CRP, an inflammatory biomarker, had a cutoff value of 0.87 mg/L with a sensitivity of 79.3% and specificity of 72.1% in this study. The AUC was 0.769 (95% CI: 0.707–0.832, *p* < 0.001). Hs-CRP is frequently considered to be a predictor of cardiovascular events. Therefore, we conclude that sPD-L1 [AUC, 0.778 (0.715–0.841); sensitivity, 73.9%; specificity, 73.4%] which exhibited results similar to those of hs-CRP [AUC, 0.769 (0.707–0.832); sensitivity, 79.3%; specificity, 72.1%], can potentially serve as a novel biomarker of disease in discriminating ACS from CAD.

## Discussion

Programmed death 1/PD-L1 inhibitors have achieved remarkable success in tumor immunotherapy, suggesting that PD-1/PD-L1 is a key pathway for inflammatory immune regulation. Recent studies have shown that this signaling pathway is also involved in the immune escape of pathogens, suggesting that PD-1/PD-L1 may play an important role in infection (30). In addition, studies using animal models of autoimmune diseases have shown that the interaction between PD-1 and PD-L1 is essential for the regulation of autoimmunity (17, 31). These data suggest that beyond their role in cancer, PD-1 and PD-L1 could be candidates for the treatment of autoimmune diseases (32). However, reports on the correlation between this pathway and the inflammatory process of coronary atherosclerosis are rare. In addition to being a possible inflammatory intervention target, soluble PD-L1 has potential value as a biomarker for tumors and other diseases, which is particularly important in coronary atherosclerosis, where there is a lack of markers related to inflammation. In this study, we first prepared a pair of antibodies with high affinity to PD-L-His protein, which were 1.80 × 10^–9^ mol/L (11E3, patent number: ZL201811063916.6) and 6.96 × 10^–9^ mol/L (2F1, patent number: ZL201811063921.7), respectively (33), and developed a very sensitive ELISA. The ELISA (Supplementary Figure S3), for which the detection limit was 0–5 pg/ml, had better sensitivity and specificity than a commercialized kit (Supplementary Figure S4). Using this ELISA method, we systematically quantified the median level of plasma sPD-L1 and found that it was 173.06 (144.81–231.50) pg/ml in normal adults and 247.03 (191.86–296.34) pg/ml in CAD patients; the highest median level of sPD-L1 in ACS patients was 256.72 (204.57–301.01) pg/ml. The current study shows that the increased levels of sPD-L1 in CAD patients, especially ACS patients, are positively related to the severity of the disease. However, in general, the increased level of sPD-L1 in ACS patients is lower than that in lung cancer patients a difference that was detected by our ELISA (unpublished data). To the best of our knowledge, there is no report of an increase in sPD-L1 levels in CAD patients; only in the course of this study did we read a recent report in which the median serum level of sPD-L1 in ACS patients was 188.7 (111.0–260.8) pg/ml and the increase in sPD-L1 levels was associated with ACS (34), which was consistent with our results.

In this study, ACS patients showed a clear increase in plasma levels of sPD-L1. It is generally believed that sPD-L1 production is usually accompanied by the expression of PD-L1 on the cell membrane surface. In addition to being expressed on tumor cells as a tumor escape mechanism, PD-L1 is highly expressed in placental and heart tissue under physiological conditions, acting as an immune barrier (11, 35). Therefore, in our opinion, there may be two main sources of sPD-L1. One is myocardial tissue. Myocardial cell injury may lead to the release of sPD-L1, and we measured high levels of sPD-L1 (data not shown) in a lung cancer patient undergoing PD-1 inhibitor treatment who developed myocardial infarction during treatment in view of the existence of myocarditis caused by immunotherapy (36). However, in our study, although multivariable logistic regression analysis revealed that sPD-L1 was significantly associated with ACS, independent of conventional coronary risk factors and other biomarkers, such as hs-CRP, BNP and hs-TnI, the linear expression analysis showed that sPD-L1 was not internally correlated with hs-TnI, which is now widely used as a gold standard for identifying patients with AMI. Therefore, our study does not support the hypothesis of a myocardial source of sPD-L1. In line with this finding, the levels of sPD-L1 in patients with ACS were still higher than those in HCs. However, whether injured cardiomyocytes increase the levels of sPD-L1 in coronary heart disease or whether the secondary inflammation caused by myocardial injury contributes to increasing the level of sPD-L1 to a certain extent needs more detailed investigation. On the other hand, this study also focused more on the correlation between the CAD inflammatory response and increased sPD-L1 levels.

The PD-1/PD-L1 pathway regulates the antitumor immune response, while signaling regulation is also associated with a substantial inflammatory response (32). PD-L1 is expressed not only on the surface of cells but also in a soluble form in the circulation, which is thought to be released from PD-L1-positive cells (37). Both soluble PD-L1 and exoPD-L1 have also been reported to be produced by PD-L1-expressing tumor cells and myeloid immune cells, such as dendritic cells (38, 39). ExoPD-L1 is a marker of adaptive immune activation (40). To evaluate the inflammatory response in CAD patients, this study systematically evaluated a panel of inflammatory cytokines covering the main stages of inflammatory response regulation. The concentrations of all 14 inflammatory cytokines were higher in CAD patients than that in HCs, indicating that patients with coronary heart disease had a systemic inflammatory response. We then compared the ACS group with the CCS and HC groups, and ACS patients showed significantly higher levels of all 14 inflammatory cytokines. Further analysis showed that the leading cytokines with increased plasma concentrations were IL-2, IL-8, IL-22, IL-6, IFN-γ, TNF-α and IL-17A, which had even higher levels in ACS patients than in CCS patients. Furthermore, only IL-6 and IL-8 were significantly associated with sPD-L1 levels in ACS patients in this study. Although IL-6 plays a central role in the inflammatory response, there has been a relative lack of evidence linking IL-6 and cardiovascular disease. Recently, a strong link between systemic inflammation and cardiovascular disease was revealed. Important evidence of a link between IL-6 and cardiovascular disease has indicated that the Asp358Ala (rs2228145, formerly known as rs8192284) variant in *IL6R* might impair classic IL-6R signaling and hence dampen inflammation, and other human genetic factors support a causal association between IL-6R-related pathways and coronary heart disease (41–44). A systematic review and meta-analysis of studies investigated the association of CRP, IL-6 and fibrinogen with the risk of recurrent stroke, and these three factors were found to be closely linked to the extent of coronary artery disease in ACS patients. Recently, higher circulating IL-6 levels in community-dwelling individuals have been shown to be linearly associated with a higher long-term risk of incident ischemic stroke, and independent of conventional vascular risk factors, they have been reported in several other studies (9, 45, 46). These reports and our results demonstrate that IL-6 is an atherothrombosis-associated inflammatory response cytokine. IL-8 is another cytokine associated with sPD-L1 levels in patients with ACS, although to a lesser extent than IL-6. In addition, there was a positive correlation between IFN-γ and sPD-L1 in UA patients. These three cytokines are associated with an increase in sPD-L1 levels, which potentially supports that sPD-L1 is a biomarker of the inflammatory response. IL-8 was initially characterized as a chemoattractant of neutrophils in acute inflammation and then discovered to be chemotactic for endothelial cells with a role in angiogenesis. IL-8 has evolved to have multifunctional activities ranging from a neutrophil chemoattractant to a promising therapeutic target for a wide range of inflammatory and neoplastic diseases, including cancer (47–49). IL-8 is produced by many cell types, including monocytes, lymphocytes, granulocytes, fibroblasts, endothelial cells, and platelets, and is released only under inflammatory conditions. Most of these cell types are abundant in atherosclerotic plaques (50). In particular, both the IL-6 and IL-8 pathways are related to macrophages, which are the core cells in coronary plaques (51). Moreover, IFN-γ can highly induce PD-L1 expression in a variety of cells, including inflammatory cells, which is considered one of the key mechanisms of tumor adaptive immune escape (52). In summary, we suggest that these cytokines are likely to support the association of sPD-Ll with the CAD inflammatory response. The increase in sPD-L1 levels in CAD patients may be the feedback involved in the regulation of the inflammatory response.

sPD-L1 has been shown to be a potential independent marker of the inflammatory response in ACS. Thus, we finally evaluated the accuracy of the established amplification ELISA in diagnosing ACS by quantifying the levels of sPD-L1 in clinical samples. Our results demonstrated that the optimum sPD-L1 cutoff for the diagnosis of ACS was 208.21 pg/ml, with a sensitivity of 73.9% and a specificity of 72.4%. The area under the curve (AUC) was 0.778. Furthermore, the optimum hs-CRP cutoff for the diagnosis of ACS was 0.87 mg/L, with a sensitivity of 79.3% and a specificity of 72.1%. The AUC was 0.769 (95% CI: 0.707–0.832, *p* < 0.001). C-reactive protein is a definitive biomarker for inflammation and has been established in clinical practice as an independent risk factor for cardiovascular disease events (38). CRP concentrations consistently increased after myocardial necrosis and coronary ischemia (39, 53). Although there was no correlation between hs-CRP and sPD-L1 in patients with ACS, the diagnostic accuracy of the two markers were comparable. There is a possibility that sPD-L1 may become a clinically useful laboratory biomarker that is different from hs-CRP, especially for patients at greatest risk for ACS. Certainly, further experimental confirmation of sPD-L1 as a new biological candidate marker is needed.

## Limitations

Although this study shows that sPD-L1 is an independent inflammatory biomarker of ACS, the possibility that false positives may be caused by infectious diseases, autoimmunity, tumors and other concomitant diseases that cause an immune response should be strictly excluded. There are other limitations, and further work is appealing and worthwhile. First, we evaluated sPD-L1 only in the plasma of CAD patients and did not assess PD-L1 on the cell surface or plaque tissues. Thus, it is difficult to reveal the whole picture and role of the PD-1/PD-L1 pathway in the pathogenesis of ACS. Second, we did not evaluate the changes in sPD-L1 levels following treatment for ACS or the association between sPD-L1 and the prognosis of CAD patients. Hence, this study could not provide therapeutic implications for patients with ACS. Third, the small number of CAD patients, especially UA and CCS patients, might provide only limited information on the levels of sPD-L1 in CAD patients, and a study with a larger sample size is needed to confirm the usefulness of sPD-L1 for risk stratification in a different population. Multicenter and prospective clinical studies with larger cohorts may be important to validate our results. Finally, previous murine studies have shown that PD-1 signaling is required to modulate the atherogenic responses of activated T cells in the arterial wall and may inhibit aggravated atherosclerosis (54). Therefore, the correlation between immune cell-based inflammatory indices and the severity of CAD is particularly important and is a priority to be explored.

## Conclusion

In general, we preliminarily found that the level of plasma sPD-L1 in patients with CAD was increased, which was positively associated with the severity of the disease and thus the progression of coronary atherosclerotic plaque. sPD-L1 is a new independently associated inflammatory biomarker that has potential diagnostic value for ACS and merits further study as a potential diagnostic target.

## Data availability statement

The raw data supporting the conclusions of this article will be made available by the corresponding author/s, without undue reservation.

## Ethics statement

The studies involving human participants were reviewed and approved by the Ethics Committee of Beijing Chest Hospital, Capital Medical University (IRB number: 2021-KY-35); the Affiliated Hospital of Jining Medical University (2022C047). The patients/participants provided their written informed consent to participate in this study.

## Author contributions

JZ and HZ contributed to the conception and design. SL, XWe, JgZ, CJ, LS, and XG contributed to the blood sampling collection and clinical data acquisition. SL, LY, XWa, ZY, BY, PW, and JW contributed to the experimental operation. JZ, HZ, and SL contributed to the analysis and interpretation of the data, writing, review, and/or revision of the manuscript. JZ contributed to the study supervision. All authors contributed to the article and approved the submitted version.

## Supplementary material

Supplementary Figure S1 Plasma levels of sPD-L1 between CAD patients and HCs of different ages. Patients between 30 and 50 years old with CAD (*n* = 29) and HCs (*n* = 39); patients between 51 and 75 years with CAD (*n* = 82) and HCs (*n* = 58). ****p* < 0.001, ***p* < 0.01. sPD-L1, soluble form of programmed cell death ligand-1; CAD, coronary artery disease; HCs, healthy controls.

Supplementary Figure S2 Association analysis of sPD-L1 with IFN-γ in UA (*n* = 22).

Supplementary Figure S3 Standard curve of the novel sandwich ELISA for sPD-L1. The capture antibody 11E3 and detection antibody Bio2F1 were followed by HRP-streptavidin to amplify the signal. The sensitivity of the detection system reached approximately 0–5 pg/ml. The error bars represent the SDs.

Supplementary Figure S4 Comparison of ROC curves between our sPD-L1 ELISA and a commercial sPDL1 kit for the diagnostic value of sPD-L1 in ACS. The area under the curve (AUC) of sPD-L1 in ACS (*n* = 76) using our sPD-L1 ELISA was 0.736, with a sensitivity of 76.3% and a specificity of 73.8%. In addition, the AUC of the commercial sPDL1 kit was 0.604, indicating a sensitivity of 60.5% and a specificity of 57.4% for the same ACS patients.

Supplementary Table S1 Baseline characteristics of 111 enrolled CAD patients.

Supplementary Table S2 The plasma levels of inflammatory cytokines in 88 CAD patients.
