# Supplementary figures and images for: Soluble programmed cell death-ligand 1 as a new potential biomarker associated with acute coronary syndrome
Source: Front Cardiovasc Med. 2022 Sep 2;9:971414. doi: 10.3389/fcvm.2022.971414 (PMC9478490; doi:10.3389/fcvm.2022.971414)

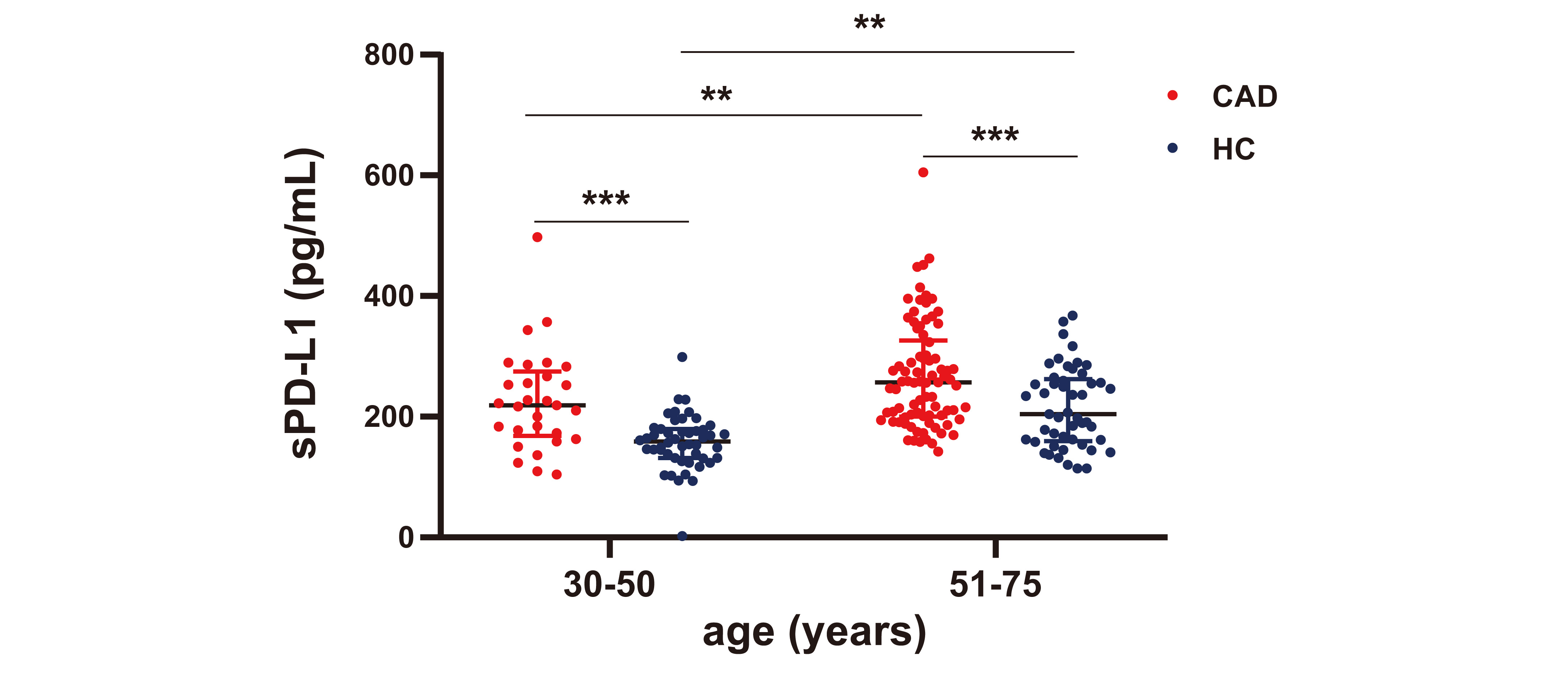

Supplement: Supplementary Figure S1 — Plasma levels of sPD-L1 between CAD patients and HCs of different ages. Patients between 30 and 50 years old with CAD (n = 29) and HCs (n = 39); patients between 51 and 75 years with CAD (n = 82) and HCs (n = 58). ***p < 0.001, **p < 0.01. sPD-L1, soluble form of programmed cell death ligand-1; CAD, coronary artery disease; HCs, healthy controls. [file Image_1.JPEG]

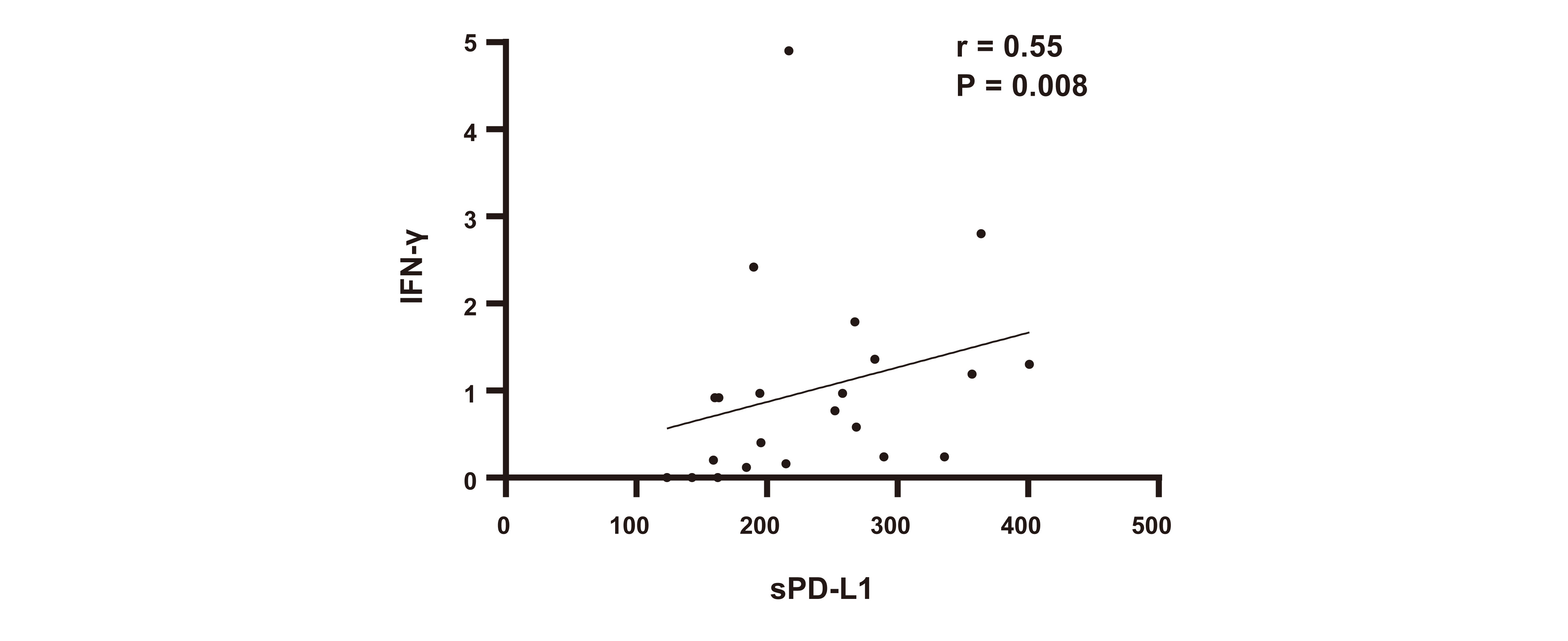

Supplement: Supplementary Figure S2 — Association analysis of sPD-L1 with IFN-γ in UA (n = 22). [file Image_2.JPEG]

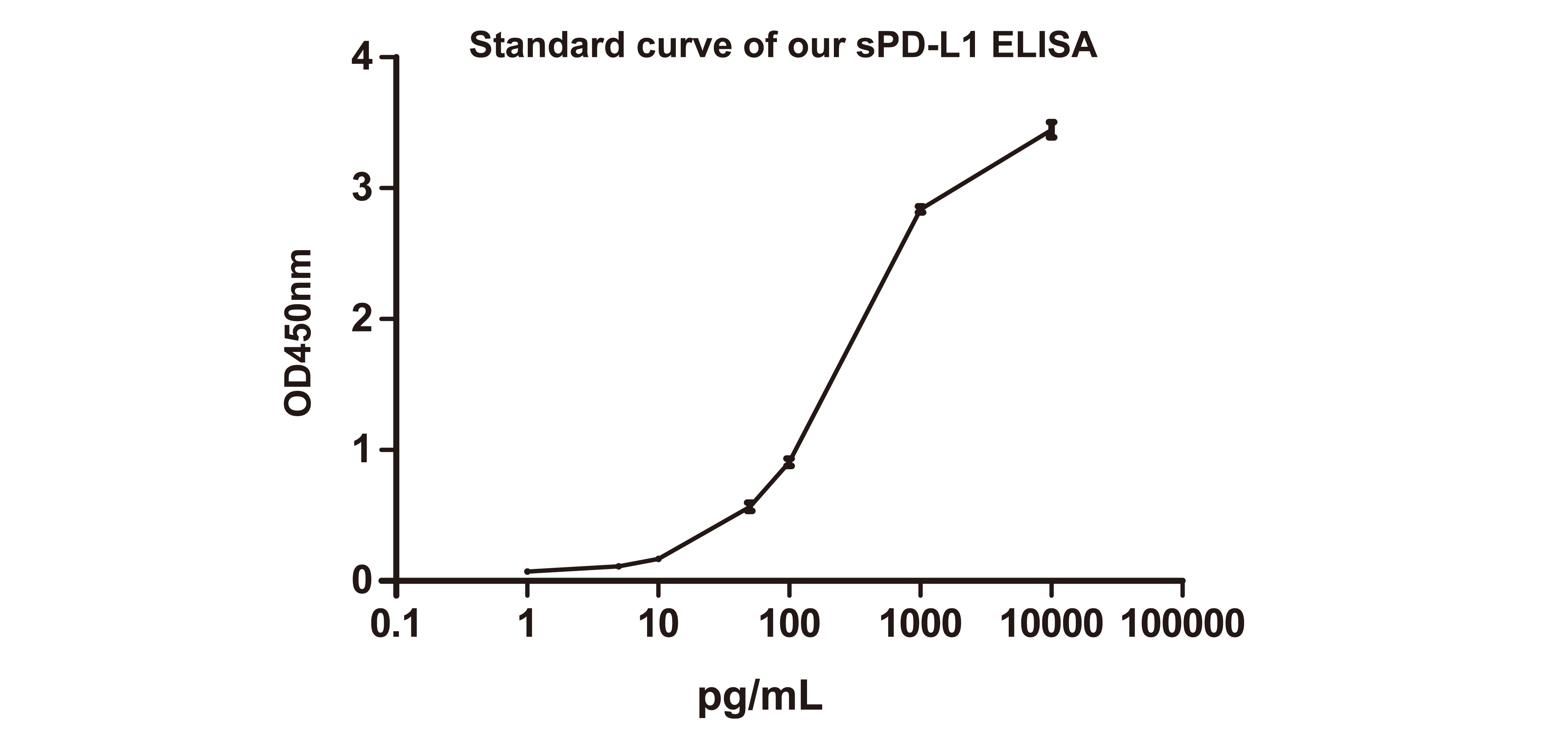

Supplement: Supplementary Figure S3 — Standard curve of the novel sandwich ELISA for sPD-L1. The capture antibody 11E3 and detection antibody Bio2F1 were followed by HRP-streptavidin to amplify the signal. The sensitivity of the detection system reached approximately 0–5 pg/ml. The error bars represent the SDs. [file Image_3.JPEG]

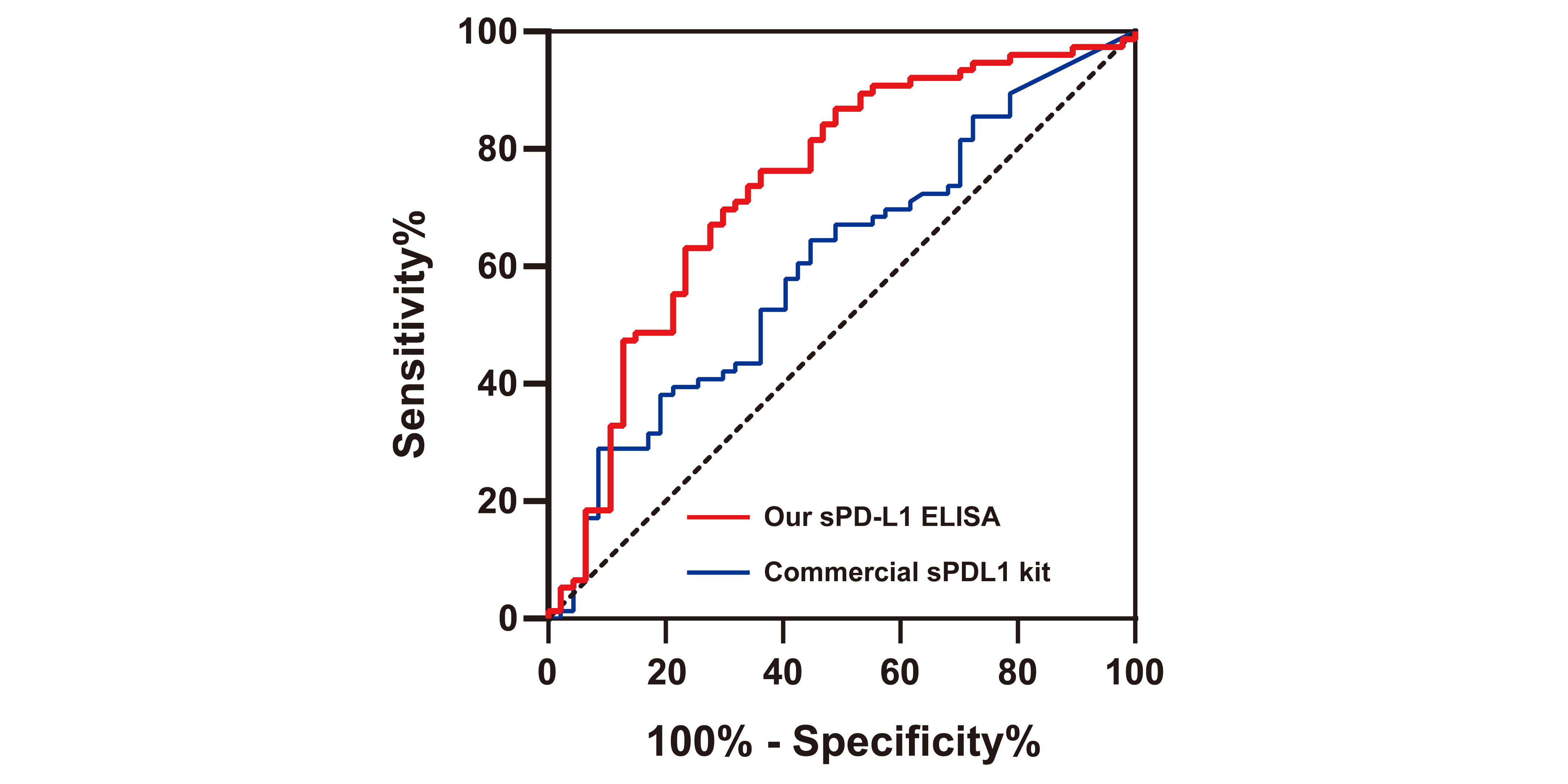

Supplement: Supplementary Figure S4 — Comparison of ROC curves between our sPD-L1 ELISA and a commercial sPDL1 kit for the diagnostic value of sPD-L1 in ACS. The area under the curve (AUC) of sPD-L1 in ACS (n = 76) using our sPD-L1 ELISA was 0.736, with a sensitivity of 76.3% and a specificity of 73.8%. In addition, the AUC of the commercial sPDL1 kit was 0.604, indicating a sensitivity of 60.5% and a specificity of 57.4% for the same ACS patients. [file Image_4.JPEG]
