# Supplementary material for: Soluble programmed cell death-ligand 1 as a new potential biomarker associated with acute coronary syndrome
Source: Front Cardiovasc Med. 2022 Sep 2;9:971414. doi: 10.3389/fcvm.2022.971414 (PMC9478490; doi:10.3389/fcvm.2022.971414)
Supplement: Supplementary Table S1 — Baseline characteristics of 111 enrolled CAD patients. [file Data_Sheet_1.PDF]

Supplementary Table 1| Baseline characteristics of 111 enrolled CAD patients.

Variable	CAD	HC	*P* value
	***N*=111**	***N*=97**	
Age, years	58.89 ± 11.60	55.21 ± 13.801	0.076
Male, n (%)	37（33.3）	69 (71.1)	0.488
BMI (kg/m2)	25.86 ± 4.28	24.54 ± 3.04	0.04
Previous MI, n (%)	9 (7.4)	0(0)	0.009
Hypertension, n (%)	80（66.1）	0 (0)	<0.001
Diabetes, n (%)	38 (31.4) *	0 (0)	<0.001
Dyslipidemia, n (%)	66 (54.5)	33 (34)	<0.001
Creatinine, μmol/L	66.8（57.60-76.90）	67 (58.6-75)	0964
Triglycerides, mg/dL	1.54（1.08-2.18）	1.29 (0.94-1.78)	0.026
HDL-C, mg/dL	1.11（0.98-1.23）	1.49 (1.16-1.71)	<0.001
LDL-C, mg/dL	2.59（2.04-3.30）	2.67 (2.25-2.98)	0.889
Hemoglobin, g/dL	136.83 ± 15.55	143.4±13.76	0.014
hs-CRP, mg/L	1.36 (0.73-3.39)	0.68 (0.42-1.57)	0.001
sPD-L1, pg/mL	247.03<br>（191.86-296.34）	173.06<br>(144.81-231.50)	<0.001

Data are presented as the n (%), mean ± SD, or median (IQR). CAD, coronary artery disease; HC, healthy control; CCB, calcium channel blockers; HDL-C, high-density lipoprotein cholesterol; LDL-C, low-density lipoprotein cholesterol; HbA1c, hemoglobin A1c, BNP, B-type natriuretic peptide; hs-CRP, high-sensitivity C-reactive protein; hs-TnI, high-sensitivity troponin I; sPD-L1, soluble programmed cell death ligand-1.
