# Supplementary material for: Soluble programmed cell death-ligand 1 as a new potential biomarker associated with acute coronary syndrome
Source: Front Cardiovasc Med. 2022 Sep 2;9:971414. doi: 10.3389/fcvm.2022.971414 (PMC9478490; doi:10.3389/fcvm.2022.971414)
Supplement: Supplementary Table S2 — The plasma levels of inflammatory cytokines in 88 CAD patients. [file Data_Sheet_2.PDF]

Supplementary Table 2 | The plasma levels of inflammatory cytokines in 88 CAD patients.

Cytokines (pg/mL)	CAD *N*=88	HC *N*=47	*p* value
IL-1β	0.96（0.86-1.18）	0.82 (0.61-0.95)	<0.001
IL-2	0.75 (0-1.4)	0 (0-0.25)	<0.001
IL-4	1.31 (0.85-2.73)	0.70 (0.31-1.26)	<0.001
IL-5	1.16(0.89-1.77)	0.77 (0.59-0.95)	<0.001
IL-6	2.07(1.48-5.30)	1.19 (0.91-1.42)	<0.001
IL-8	2.7(1.25-4.13)	1.85 (1.13-2.48)	0.013
IL-10	1.52(1.23-2.12)	1.02 (0.82-1.31)	<0.001
IL-12p70	1.04(0.91-1.22)	0.74 (0.63-0.82)	<0.001
IL-17A	0.91(0.46-1.64)	0.2 (0-0.62)	<0.001
IL-17F	0.93(0.73-1.42)	0.50 (0.43-0.61)	<0.001
IL-22	0.61(0.3-1.22)	0.13 (0.06-0.21)	<0.001
TNF-α	0.61(0.28-1.37)	0.43 (0.3-0.64)	0.018
TNF-β	2.47(2.07-2.96)	1.62 (1.52-1.72)	<0.001
IFN-γ	0.77(0.25-1.79)	0 (0-0.08)	<0.001

Data are presented as the median (IQR). ACS, acute coronary syndrome; CCS, chronic coronary syndrome; HC, healthy control; interleukin, IL; TNF-α, tumor necrosis factor alpha; TNF-β, tumor necrosis factor beta; IFN-γ, interferon gamma.
